# p53 Functions in Adipose Tissue Metabolism and Homeostasis

**DOI:** 10.3390/ijms19092622

**Published:** 2018-09-04

**Authors:** Jelena Krstic, Isabel Reinisch, Michael Schupp, Tim J. Schulz, Andreas Prokesch

**Affiliations:** 1 Gottfried Schatz Research Center for Cell Signaling, Metabolism & Aging, Department of Cell Biology, Histology and Embryology, Medical University Graz, 8010 Graz, Austria; jelena.krstic@medunigraz.at (J.K.); isabel.reinisch@medunigraz.at (I.R.); 2 Berlin Institute of Health, Institute of Pharmacology, Center for Cardiovascular Research, Charité-Universitätsmedizin Berlin, Corporate Member of Freie Universität Berlin and Humboldt-Universität zu Berlin, 10117 Berlin, Germany; michael.schupp@charite.de; 3 Department of Adipocyte Development and Nutrition, German Institute of Human Nutrition, 14558 Potsdam, Germany; Tim.Schulz@dife.de; 4 German Center for Diabetes Research (DZD), München-Neuherberg, 85764 Neuherberg, Germany; 5 Institute of Nutritional Science, University of Potsdam, Potsdam-Nuthetal, 14558 Nuthetal, Germany; 6 BioTechMed-Graz, 8010 Graz, Austria

**Keywords:** p53, adipose tissue, metabolic syndrome, obesity, adipogenesis, insulin resistance

## Abstract

As a tumor suppressor and the most frequently mutated gene in cancer, p53 is among the best-described molecules in medical research. As cancer is in most cases an age-related disease, it seems paradoxical that p53 is so strongly conserved from early multicellular organisms to humans. A function not directly related to tumor suppression, such as the regulation of metabolism in nontransformed cells, could explain this selective pressure. While this role of p53 in cellular metabolism is gradually emerging, it is imperative to dissect the tissue- and cell-specific actions of p53 and its downstream signaling pathways. In this review, we focus on studies reporting p53’s impact on adipocyte development, function, and maintenance, as well as the causes and consequences of altered p53 levels in white and brown adipose tissue (AT) with respect to systemic energy homeostasis. While whole body *p53* knockout mice gain less weight and fat mass under a high-fat diet owing to increased energy expenditure, modifying *p53* expression specifically in adipocytes yields more refined insights: (1) p53 is a negative regulator of *in vitro* adipogenesis; (2) p53 levels in white AT are increased in diet-induced and genetic obesity mouse models and in obese humans; (3) functionally, elevated p53 in white AT increases senescence and chronic inflammation, aggravating systemic insulin resistance; (4) p53 is not required for normal development of brown AT; and (5) when p53 is activated in brown AT in mice fed a high-fat diet, it increases brown AT temperature and brown AT marker gene expression, thereby contributing to reduced fat mass accumulation. In addition, p53 is increasingly being recognized as crucial player in nutrient sensing pathways. Hence, despite existence of contradictory findings and a varying density of evidence, several functions of p53 in adipocytes and ATs have been emerging, positioning p53 as an essential regulatory hub in ATs. Future studies need to make use of more sophisticated *in vivo* model systems and should identify an AT-specific set of p53 target genes and downstream pathways upon different (nutrient) challenges to identify novel therapeutic targets to curb metabolic diseases.

## 1. Introduction

The tumor suppressor p53 has been attributed a central role in cancer research mostly due to the fact that more than half of all human cancers present with a mutation in the *TP53* gene [1]. Accordingly, it has been tagged with monikers like “guardian of the genome” or “master cell cycle regulator” [2] and was coined the top investigated gene by the end of 2017 [3]. There is a plethora of evidence that p53 regulates biological processes like the stress response, cell cycle, proliferation, invasion, senescence, apoptosis, and autophagy; to name only the most important ones [2,4]. In addition, noncanonical functions of p53 in post-mitotic, noncancerous cells have been described more recently [5,6,7]. These cancer-unrelated functions of p53 are plausible with respect to the process of evolutionary selection, because cancer is a disease that appears, or becomes life-threatening, mainly after reproductive age. It follows then that the strong degree of conservation, which implies a selective advantage, can only be explained by tumor suppressor-independent functions in nontransformed cells and tissues [8]. In the case of p53, these functions include cell fate decisions (stem cell maintenance and pluripotency) and manipulation of cell metabolism and energy homeostasis (involvement in anabolism and catabolism of all macronutrient classes). How one transcription factor can cover such a multitude of context-specific, diverse, and comprehensive cellular processes is only partially understood. Gaining knowledge of the exact regulatory in- and output of signaling that pertains to wild-type p53 and its many known mutant forms in specific cells, organs, and during physiological and pathological situations, comprises both a major challenge and hope for the implementation of personalized medicine in the clinic.

Adipose tissue consists of locally and functionally distinct depots with different cellular makeups [9,10,11], including nonparenchymal cells like endothelial cells, stem and progenitor cells, and various immune cells [12]. White adipose tissue (WAT) depots are mainly found in the abdominal and subcutaneous regions, while brown adipose tissue (BAT) is found interscapular (in human neonates and rodents) or in smaller depots distributed throughout the upper torso [13]. Both WAT and BAT consist of a mixture of brown, beige (brown-like), and white fat cells, but BAT depots are much more vascularized and innervated compared to pure WAT depots [13] because of its importance for cold-induced, nonshivering thermoregulation [14,15,16]. White adipocytes are specialized in storing fatty acids (FAs) in the form of triglycerides and release them through lipolysis during times of nutrient deprivation or increased metabolic demand [17]. Via secreting metabolites, adipokines (e.g., leptin, adiponectin, resistin [18], or cytokines such as IL-6 [19]), and exosomal microRNAs [20,21] adipose tissue depots communicate with other tissues and the immune system to coordinate whole body energy homeostasis [22]. Because adipose tissue is a major integrator of whole body energy homeostasis, its manipulation has become a promising strategy in the fight against obesity and its associated diseases (i.e., metabolic syndrome). In this context, BAT activation and heat dissipation of energy or WAT features such as expandability, endocrine function, and inflammation have been targeted for therapeutic intervention [23]. In addition to its contribution to the development of the metabolic syndrome, obesity shows a strong association with cancer [24,25]. Hence, tumor suppressors that contribute to regulation of cellular metabolism could present an important connection linking obesity and cancer. Paradoxically, despite *p53* being frequently mutated in liposarcoma [26,27] and its involvement in adipose tissue biology, the incidence rate of liposarcoma is very low if compared to other cancers that arise through p53 loss-of-function [1].

p53’s general implication in the regulation of lipid and lipoprotein metabolism has been reviewed before [28]. In this review, we summarize the current literature on the functions of p53 in adipocyte development and in adipose tissue homeostasis. Because this topic has been reviewed in 2009 [29,30], we attempt to highlight more recent findings. Furthermore, we try to evaluate studies exploring manipulation of p53 levels in adipose tissue depots and the impact on systemic energy metabolism in the context of insulin resistance and obesity.

## 2. p53 Signaling during In Vitro Adipogenic Differentiation

The potential of WAT to store excess lipids as triglycerides has major implications on whole body physiology and pathophysiology, not only by providing energy substrates in times of nutrient scarcity but also by protecting the other organs from lipotoxicity [31,32]. Subcutaneous adipose tissue in humans shows a low cellular turnover rate of only about eight percent per year [33]. Furthermore, the increase in fat mass, when energy intake exceeds energy expenditure, is mainly due to hypertrophy rather than hyperplasia [34]. Despite these facts, the differentiation capacity of adipogenic precursor cells contributes to the pathogenic phenotype seen in many obese subjects. This was supported by studies showing that loss of adipogenic differentiation potential with advanced age is accompanied by accumulation of lipids outside of adipose tissue (summarized previously [35]).

Adipocytes derive from the mesenchyme and the adipogenic program can roughly be divided into two major phases. In the first phase, mesenchymal stem cells commit to the adipogenic lineage (determination phase) and are then termed preadipocytes. In the second phase (terminal differentiation phase), these preadipocytes acquire the characteristics of mature adipocytes [22]. The terminal differentiation phase is regulated by a complex transcriptional cascade [36]. Major regulators are the nuclear receptor peroxisome proliferator-activated receptor gamma (Pparγ) that is necessary and sufficient for adipogenesis, and the basic leucine zipper transcription factors of the CCAAT/enhancer binding protein (C/EBP) family [37,38,39,40]. Several other factors linked to adipogenesis have been described, including transcriptional coactivators and corepressors [41] and signaling pathways [37]. While p53 is involved in the differentiation of hematopoietic as well as nonhematopoietic cells [42], a detailed picture of p53’s mode of action during the adipogenic differentiation program has yet to emerge, as discussed below.

Despite the availability of specialized mouse models, such as the FAT-ATTAC-mouse in which induced apoptosis of mature adipocytes in adult mice is followed by differentiation from precursors [43], studying adipogenesis *in vivo* is complicated by the absence of preadipocyte markers and by differences in adipogenic capacity of different WAT depots [44]. However, there are various models to study adipogenesis *in vitro*. In the murine preadipocyte cell line, 3T3-L1, one of the standard models for *in vitro* adipogenesis, nutlin-3a-mediated p53 protein accumulation as well as ectopic *p53* overexpression led to a decrease in the expression of *Pparγ* and of its coactivator Pparγ coactivator1 alpha (*Pgc1α* or *Ppargc1a*) [45,46,47]. The knockdown of *p53* in differentiated 3T3-L1 cells led to enhanced *Pgc1α* mRNA expression and Pparγ2 and fatty acid binding protein 4 (Fabp4) protein levels [45,46], as well as to accelerated accumulation of lipid droplets [46]. Although these results suggest that p53 is a negative regulator of the adipogenic program, data about p53 signaling in 3T3-L1 cells need to be interpreted with caution, since the transformed mouse 3T3 cell double minute 2 (*Mdm2*) gene, whose encoded protein is the major negative regulator of p53, is highly amplified in this cell line [48]. Therefore, p53 signaling in 3T3-L1 cells could be constitutively altered. The multipotent C3H10T1/2 mouse cell line possesses the ability to differentiate into white adipocytes in addition to several other lineages upon appropriate induction [49,50]. In comparison to 3T3-L1 cells, this cell line can be used to study earlier events of adipogenesis [51]. Knockdown of *p53* in C3H10T1/2 cells led to enhanced differentiation with upregulation of adipogenic genes throughout the differentiation process [45]. Also, upon adipogenic induction of the multipotent mouse stromal MBA-15 cell line, knockdown of *p53* led to increased levels of *Pparγ*, *Fabp4*, and adiponectin (*Adipoq*) and increased adipocyte colony formation [52]. Similar results were reported in human adipose tissue-derived stem cells [45]. As an intermediate between immortalized cell lines and *in vivo* studies, mouse embryonic fibroblasts (MEFs) were isolated from *p53* knockout mice and differentiated into adipocytes [46,52] that accumulated more lipid droplets and showed a moderate increase in adipogenic markers (*Pparγ*, *C/EBPα*) compared to wild-type MEFs. Importantly, *p53* knockout MEFs underwent spontaneous commitment to the adipogenic program and ectopic re-expression of *p53* in knockout MEFs efficiently inhibited their spontaneous differentiation [52,53]. Moreover, nutlin-3a-mediated p53 accumulation in wild-type MEFs led to downregulation of *Pparγ* [52]. This negative role of p53 in adipocyte differentiation in cell lines and MEFs is supported by data from primary cells derived from the stromal vascular fraction of inguinal WAT or of interscapular BAT from *p53* knockout mice [54] and by results from human adipocyte-derived stem cells with *p53* knockdown [45], as in both systems p53 reduction led to increased differentiation potential. Looking into possible mechanisms of the negative effect of p53 on adipogenesis, Hallenborg et al. suggested that the ability of p53 to inhibit adipogenesis is dependent on its binding to DNA, because introducing mutations to the DNA-binding domain of *p53* failed to inhibit adipocyte conversion of wild-type MEFs [53]. These results suggest that the inhibitory function of p53 in adipogenesis is due to its transcriptional activity. Corroborating this, MEFs deficient for *Cdkn1a*, encoding for a canonical transcriptional target of p53, the cyclin-dependent kinase inhibitor 1a (also known as p21^WAF1/CIP1^ or p21 [55,56]), underwent spontaneous adipose conversion, as shown by oil red O staining and adipocyte marker gene expression [53]. *In vivo* studies showed that knockout of *p21* in mice leads to adipocyte hyperplasia [57]. Contradictory, Inoue et al. showed that the knockdown of *p21* leads to impaired adipocyte differentiation of 3T3-L1 cells and MEFs [58]. However, effects in the study of Inoue et al. were observed in the terminal differentiation phase of the adipogenic program, whereas the anti-adipogenic effects were evident in the earlier determination phase of adipogenesis.

Coactivator-associated arginine methyltransferase 1 (Carm1), that has been shown to enhance adipogenesis by activating Pparγ [59], was recently suggested as a possible mediator of the anti-adipogenic effects of p53 [60]. The authors found that p53 bound to and repressed the *Carm1* promoter. Furthermore, p53 activation by nutlin-3a treatment in 3T3-L1 cells led to a reduced adipocyte phenotype and a concomitant downregulation of Carm1 mRNA and protein. Importantly, *Carm1* overexpression rescued the reduction of adipocyte differentiation by nutlin-3a treatment [60].

Summarizing data from different *in vitro* adipogenesis systems, there is strong evidence that p53 negatively regulates the adipogenic program possibly by impinging on central regulatory hubs of early adipogenic differentiation. However, more work is needed to define the exact network of p53 interactions responsible for its anti-adipogenic function. Furthermore, due to a lack of data from *p53* knockout in mature adipocytes it remains unknown whether p53 is involved in adipocyte maintenance and nutrient homeostasis.

## 3. p53 in Obesity and Adipose Tissue Insulin Resistance

Obesity develops due to an imbalance between calorie intake and expenditure, which can be caused by a variety of factors ranging from behavioral to genetic, resulting in increased visceral adiposity and, in most cases, systemic low grade chronic inflammation [61]. Obesity is a major determinant for the metabolic syndrome and predisposes patients to insulin resistance and type 2 diabetes mellitus, cardiovascular disease, and cancer [25,61]. Insulin maintains glucose homeostasis by inhibiting glucose production (gluconeogenesis) in the liver and stimulating glucose uptake in peripheral tissues, such as adipose tissue and skeletal muscle [62,63]. Given the wide range of insulin target tissues, insulin resistance impairs glucose homeostasis in the whole body. This confounds the aim to establish the role of single tissues in insulin resistance. However, the endocrine function of adipose tissue, entailing the production of hormones and other regulatory proteins, which can affect the immune system and the activity of other tissues and organs (vascular system, heart, pancreas, etc.) [64], nominates it as a central regulator of insulin resistance together with muscle and liver.

First evidence about p53’s involvement in obesity came from a study done in genetically obese (*ob*/*ob*) mice and showed p53 mRNA and protein induction in epididymal WAT of these animals, which correlated inversely with sterol regulatory element-binding transcription factor 1 (*Srebf1*) mRNA expression. This, and luciferase reporter assays on the *Srebf1* and fatty acid synthase (*Fasn*) promoter, led the authors to conclude that p53 represses the lipogenic Srebf1 pathway in adipocytes [65]. Following this initial finding, p53 mRNA and/or protein induction in visceral WAT of obese mice [66,67,68,69], rats [70], and humans [71,72] was observed. While its induction in obesity was reproduced in many studies, the mechanisms of p53 activation and its role on adipocyte function remain inconclusive, although a significant amount of research has been done in this field.

Tissue senescence is linked to impaired immune responses, leading to a chronic inflammatory state observed in both obesity and aging [73]. Obesity can be perceived as an accelerated form of adipose tissue senescence, linked to comorbidities similar to those attributed to aging (e.g., diabetes, heart failure, and cancer [73]). Regulation of cellular senescence is one of the canonical functions of p53 [4]. Minamino et al. demonstrated that genetically hyperphagic and obese *Ay* mice display increased p53 protein levels, increased reactive oxygen species (ROS) abundance, enhanced activity of senescence-associated β-galactosidase, and increased expression of genes encoding pro-inflammatory cytokines (such as tumor necrosis factor (*Tnf*) and chemokine (C-C motif) ligand 2 (*Ccl2*)) in epididymal WAT, as well as insulin resistance [71]. Heterozygous whole body *p53* deletion in *Ay* mice resulted in the reduction of both senescence and inflammation, with insulin resistance being almost completely abolished [71]. Of note, fat tissue weight only slightly decreased in *Ay Trp53*^+/−^ mice, implying that a functional change in adipose tissue, rather than altered hypertrophy or hyperplasia, was responsible for improvements of insulin resistance. The insulin resistant phenotype and p53 induction similar to that of *Ay* mice was also observed in mice with dietary obesity fed a high-fat, high-sucrose (HFHS) diet [71]. Adipose tissue-specific *p53* knockout (via Fabp4-Cre system) in these mice also resulted in the reversal of the insulin resistant phenotype as well as normalized hepatic gluconeogenic enzyme expression (which were increased in *Ay* and diet-induced obese mice), without modifying the visceral WAT weight. Furthermore, *p53* overexpression in adipose tissue led to increased insulin resistance [71]. This study outlined a relevant role of p53 in insulin resistance development via senescence induction in visceral WAT. Building on the aforementioned observations it would be of relevance to investigate how adipose tissue senescence regulated by p53 drives chronic inflammation in visceral WAT and beyond. A follow-up study in mice investigating the mechanistic link between p53 and inflammation onset discovered that the semaphorin 3E (Sema3e)-plexin D1 axis mediates inflammation orchestrated by p53 induction in visceral WAT during dietary obesity [67]. Using several elegant *in vivo* approaches, it was demonstrated that p53 induces upregulation of Sema3e which acts as chemoattractant for macrophages in visceral WAT [67].

Although upregulation of both p53 activity and inflammation is commonly observed in WAT of obese rodents and humans, the causal relationship remains unclear. A positive correlation between *p53* expression, inflammation (e.g., *TNF* gene expression and CD68-positive macrophage infiltration) and body mass index (BMI) was observed in the omental (and not in subcutaneous) WAT depot in a study involving a cohort of 230 human subjects [72]. In support of inflammation-induced p53 activity postulated by the authors, *in vitro* experiments showed that the anti-inflammatory effects of endogenously produced adiponectin or of rosiglitazone treatment reduced *p53* expression in differentiated adipocytes. In line, addition of LPS-treated macrophage-conditioned medium increased *p53* expression in differentiated adipocytes [72]. Importantly, Minamino et al. also analyzed visceral WAT from nondiabetic and diabetic human subjects and showed increased senescence, p53 induction, and increased *TNF* and *CCL2* expression in diabetic patients [71]. Opposing this finding, Ortega et al. found that *p53* mRNA was inversely associated with glucose intolerance in human non-obese or obese subjects [72]. These seemingly contradictory results may be due to p53’s diverse functions (and levels) in non-obese and obese subjects. As shown in lean mice, p53 functions as a regulator of glucose homeostasis and its downregulation leads to impaired glucose tolerance (see chapter “Nutritional regulation of p53 in adipose tissue”) [74,75]. While this finding remains to be confirmed in humans, one could speculate that in obese subjects, duration of p53 elevation could be a contributing factor determining the difference between the glucose-tolerant and glucose-intolerant subjects. However, as chronic adipose tissue p53 elevation is a phenomenon that seems impossible to measure, this will likely remain a plausible, but hard-to-prove, hypothesis.

Parallel to p53 induction, reduced expression of *MDM2* was observed in human omental WAT [72], implying a disturbed MDM2-mediated p53 regulation in obesity. Mice harboring a mutation in *Mdm2* (*Mdm2^C305F^*), which results in higher availability of Mdm2 due to disrupted binding to ribosomal protein L11 (Rpl11) and consequently higher ubiquitination and reduced activity of p53, are resistant to high-fat diet (HFD)-induced obesity [66]. Reduction of p53 activity in *Mdm2^C305F^* mice resulted in lower adiposity, improved glucose tolerance, increased energy expenditure, and reduced inflammatory cytokine gene expression in epididymal WAT as well as in reduced liver steatosis [66]. These findings were confirmed in *p53*^+/−^ mice. However, when fed normal chow, *Mdm^2C305F^* mice are phenotypically and metabolically comparable to wild-type mice. The authors proposed that the Rpl11-Mdm2-p53 axis has a central role in energy expenditure in response to nutrient availability [66].

p53 was also assigned a central position in the connection between cardiac dysfunction and insulin resistance [68]. Introduction of transverse aortic constriction (TAC) which induces pressure overload, caused inflammation in adipose tissue, increased insulin resistance, and reduced glucose tolerance in mice. Concomitantly, lipolysis was induced and release of FAs led to chronic p53 upregulation, detectable in visceral WAT as long as six weeks after TAC [68]. When TAC was applied in adipose tissue-specific *p53* knockout mice (using Fabp4-Cre/lox system), inflammation in visceral WAT was reduced and insulin sensitivity and glucose tolerance were improved. However, TAC in adipose tissue-specific *p53* knockout mice did not result in lower visceral WAT weight or plasma FA levels compared to wild-type control mice, indicating that the increase in lipolysis is independent of p53 induction [68]. Furthermore, lipolysis was induced along with p53 in isoproterenol-treated epididymal fat pads isolated from wild-type mice and disruption of lipolysis in adipose tissue isolated from adipose triglyceride lipase (*Atgl*) knockout mice resulted in lower p53 protein level, indicating that increased free FA levels are responsible for p53 induction in visceral WAT. Additional *in vitro* experiments confirmed p53 induction upon treatment with palmitic acid [68].

In addition to mediating insulin resistance in the background of diet-induced obesity, p53 was also shown to mediate insulin resistance caused by excess growth hormone (Gh) [76]. Upregulation of p53 both in diet-induced obese mice and *Acro* mice (transgenic mice overexpressing bovine growth hormone gene) was restricted to WAT, and no difference in the p53 protein levels was noted in liver, kidney, or skeletal muscle. A similar degree of insulin resistance and glucose intolerance was observed in both mouse models, effects which were reversed after p53 inhibition by pifithrin or Gh inhibition by pegvisomant, both treatments that reduced p53 levels in adipose tissue [76]. Impaired glucose uptake by adipose tissue, due to reduction in the glucose transporter solute carrier family 2 member 4 (Slc2a4 or Glut4) and increase in Srebf1 were shown in the same study. It was therefore suggested that p53 is a central regulator of insulin resistance driven by increased sensitivity of adipose tissue to Gh. This was suggested to be mediated by suppressors of cytokine signal (Socs) and signal transducers and activators of transcription (Stat) proteins acting upstream of p53 and resolved via decreased glucose uptake (by Glut4 downregulation) and glycolysis inhibition (by Trp53 induced glycolysis regulatory phosphatase (Tigar) upregulation and hexokinase (Hk2) downregulation), as well as by impaired lipid metabolism due to decreased FA uptake via Sreb1 downregulation [76]. However, these results rely on the activity of inhibitors with potential off-target effects, as already observed for pifithrin [77,78].

Perhaps the most comprehensive study of p53’s role in the etiology of obesity and insulin resistance considered the temporal and causative component of the events occurring in obesity development [69]. By using diet-induced obese mice, primary adipocytes, and the 3T3L1 cell line, Vergoni et al. showed that increase in ROS and DNA damage in visceral WAT occur together with upregulation of p53, p21, and cyclin dependent kinase inhibitor 2A (*Cdkn2a* or *p16Ink4a*) as early as 2–4 weeks of feeding a HFD. At this stage, lipogenesis was slightly induced while inflammation was increased only after 18 weeks of HFD. Insulin resistance was also observed after 18 weeks and not present in mice after 2 weeks on HFD. Short-term treatment of 3T3-L1 adipocytes with p53-inducers doxorubicin and nutlin-3a resulted in increased expression of inflammatory cytokine/chemokine mRNA and in the reduction of insulin-stimulated glucose uptake by reduced Glut4 glucose transporter at the cells’ plasma membrane. Furthermore, conditioned media from the same 3T3-L1-treatments acted as a chemoattractant, inducing *in vitro* macrophage and neutrophil migration [69]. Of note, DNA damage was also observed in the adipose tissue of *Ay* mice and telomerase reverse transcriptase (*Tert*) deficient mice [71]. Vergoni et al. showed that the immediate increase in inflammation upon p53 induction observed *in vitro* [69,72] does not necessarily correlate with the sequence of events observed in intact tissues (at least in mice), where inflammation does not precede p53 induction.

The impact of the *p53* mutations on whole body metabolism was investigated in humanized p53 mice in which the mouse *p53* locus was replaced by the human gene variant, harboring either a proline (P72) or an arginine (R72) on position 72 of the amino acid chain (rs1042522). In humans, these two *p53* variants represent an allele frequency distribution that depends on geographical latitude (P72 is predominant in equatorial regions and R72 is predominant in the northern hemisphere [79]). Interestingly, while R72 mice are phenotypically similar to P72 mice when fed a normal chow diet [80], under HFD the R72 variant-carrying mice exhibit increased weight gain, concomitant with increased fat mass, adipose tissue immune cell infiltration, hepatic steatosis and fibrosis, and insulin resistance [81]. These prediabetic conditions in R72 mice were attributed to deregulation of p53 early response target genes NPC1 like intracellular cholesterol transporter 1 (*Npc1l1*) and *Tnf* in the liver, because systemic pharmacological inhibition of these targets blunted the weight gain under HFD. The question whether p53 action in adipose tissue contributes to the metabolic phenotype of R72 mice warrants further experimental attention, especially because genome-wide association studies indicated a strong association of the R72 variant with increased BMI [81,82] and the prevalence of type 2 diabetes in humans [83,84,85].

Current knowledge on p53’s role in adipose tissue is mostly based on its activity in adipocytes in visceral WAT as the most metabolically active adipose depot. In addition to the different WAT depots with distinct and linked metabolic functions, each of the depots is comprised of a number of cell types. In general, WAT consists mostly of adipocytes, but also contains pre-adipocytes, mesenchymal stem cells, fibroblasts, immune, and endothelial cells [12]. There is a lack of information about p53 in nonparenchymal, adipose-resident cells. For instance, in the light of results demonstrating that p53 regulates M2 macrophage polarization [86], it would be of special importance to investigate the function of p53 in adipose tissue-resident macrophages, as they are the most abundant immune cell type in obesity-related inflammation. The strong influence of endothelial p53 on the development of obesity and related metabolic abnormalities has been demonstrated in research conducted in mice in which *p53* was knocked out specifically in endothelial cells. Under a HFHS diet, *p53* loss led to reduced body weight, reduced adiposity (both in visceral WAT and subcutaneous WAT), lowered inflammation, and improved insulin sensitivity and glucose tolerance when compared to wild-type mice [87]. Furthermore, overexpression of *p53* in endothelial cells resulted in opposite outcomes in HFD-fed mice. The described effects in endothelial cell-specific *p53* knockout mice were attributed to increased energy expenditure and glucose uptake in skeletal muscle [87], although the study was not focusing on adipose-resident epithelial cells. Investigation of p53 expression and function in adipose tissue-resident, nonparenchymal cell types would contribute to the clarification of p53’s complex role in adipose tissue metabolism.

p53 is generally considered as a stress response protein. The experimental conditions during investigations of p53’s role in obesity or insulin resistance development can be interpreted as different stress circumstances that elicit a p53 response. The consensus observation is that chronic p53 induction takes place in visceral WAT in response to metabolic stressors, such as caloric overload (by HFD or HFHS diet), aging or cellular senescence, or cardiac dysfunction (induced by TAC or myocardial infarction) [68]. Furthermore, like in diet-induced obese mice [66,67,69,71], induction of p53 has been observed in visceral WAT of *ob*/*ob* mice [65] as well in endothelial cells of HFHS-fed mice [87]. In the absence of stressors, p53 is necessary for glucose homeostasis [74,75]. Even though induction of p53 protein in visceral WAT of obese mice is well supported by evidence, neither the cause nor the effect of this upregulation is completely understood, similar to the situation in liver insulin resistance [7]. Still, some pieces of the puzzle have been unraveled: p53 can be induced by ROS or FFAs in adipocytes and visceral WAT; p53 induction can lead to visceral WAT senescence and inflammation onset (e.g., via upregulation of target genes like *Sema3E* that promotes the attraction of macrophages); this is then further fueling the adipose tissue stress response via p53 in a potential feedback loop.

## 4. Nutritional Regulation of p53 in Adipose Tissue

p53’s involvement in metabolic homeostasis includes the metabolic adaption to situations of nutrient deprivation or overabundance [6,88,89]. The systemic response to nutrient availability is mainly orchestrated by the liver [90,91]. p53 in the context of liver diseases and metabolism has been previously reviewed by us [7]. Therefore we here briefly summarize the main points on nutritional regulation of p53 in liver and emphasize on effects in adipose tissue.

In mice with normal calorie intake p53 seems mainly involved in glucose homeostasis. Mice harboring a germ line mutation in the p53 phosphorylation site Ser18 (a binding site of ATM protein kinase) exhibit defects in p53-mediated apoptosis and transcriptional activity [74]. When maintained on normal chow, *p53S18A* mice display insulin resistance and increased serum inflammation markers. One possible explanation for these observations could be that accumulation of oxidative damage via downregulation of sestrin genes (*Sesn1* and *Sesn2*) in the absence of functional p53 leads to impaired glucose homeostasis [74]. Similarly to *p53S18A* mice, *p44Tg* mice, harboring a p53 isoform which lacks the transactivation domain and competes with full length p53 resulting in its reduced transcriptional activity, exhibited similar defects in glucose homeostasis, as well as reduced epididymal WAT expression of adipose tissue marker genes [75]. On the other hand, ‘super p53’ mice that harbor additional alleles of *p53* exhibited improved glucose tolerance when fed normal chow diet [75]. In *p53S18A* and *p44Tg* mice, a decrease in p53 activity led to a slight increase in body weight, although WAT weight was not investigated [74,75]. Furthermore, insulin resistance occurred in middle-aged mice, implying that chronic reduction of p53 activity is responsible for the development of insulin resistance under normal calorie intake. However, due to modulation of p53 activity in the whole body, it is difficult to assign these metabolic phenotypes specifically to liver, or adipose tissue, or any other tissue. It is likely that accumulative and/or synergistic effects promote the reported metabolic alterations.

Under excess calorie intake, p53 induction in visceral WAT occurs along with inflammation and insulin resistance, as described in more detail in the previous chapter. The effects of HFD on p53 induction are usually monitored after the onset of obesity (6–20 weeks on HFD), except in one study which showed p53 induction in visceral WAT after 2 weeks of HFD [69]. However, experimental abrogation of p53 in obese mice did not always result in the reduction of body weight or visceral WAT [71]. A spectrum of different animal models with different genetic backgrounds along with variation in used diets and treatment durations are a reasonable explanation for such discrepancies.

A central regulatory role of the ribosomal protein-Mdm2-p53 axis has been exposed for energy expenditure and lipid storage in response to nutrient availability [66,92]. Liu et al. showed that p53 reduction in the liver of *Mdm2^C305F^* mice resulted in defective free FA transport into mitochondria and oxidative phosphorylation, thereby compromising liver lipid homeostasis during starvation [92]. On the other hand, nutrient abundance caused Rpl11-dependent p53 activation, and reduced expression of genes associated with energy metabolism (such as *Pgc1α*, *Ppargc1b*, *Glut4*, and sirtuin 1 (*Sirt1*)), resulting in reduced NAD^+^ levels and energy expenditure, ultimately leading to obesity. Disruption of Rpl11 binding by mutated Mdm2 blocked HFD-induced p53 activation and led to increased energy expenditure and improved resistance to the development of obesity [66]. The authors hypothesize that the p53 response to nutrient availability represents a conserved stress response needed for survival, which is causing unfavorable effects (e.g., obesity) in modern societies where nutrients are chronically abundant [66,93].

Our own data confirmed the essential role of liver p53 under starvation: To adapt to nutrient scarcity in starvation conditions, various transcription factors are activated, which regulate genes involved in metabolic pathways that ensure homeostasis, most importantly via gluconeogenesis and ketogenesis [94,95]. We found that p53 target genes are elevated in liver, epididymal WAT, and skeletal muscle after 24 h of food withdrawal [96]. Additionally, liver-specific *p53* knockout mice showed defects in amino acid metabolism and hypoglycemia under starvation [97]. In support of the progluconeogenic role of p53 are findings showing that p53 promotes the expression of genes encoding proteins involved in gluconeogenic precursor supply and gluconeogenesis [98] and that by knocking out pantothenate kinase 1 (*Pank1*), which is a direct transcriptional target of p53, β-oxidation and gluconeogenesis are negatively affected in the liver of mice under starvation [99]. Contradictory, Zhang et al. reported that hepatic p53 represses gluconeogenesis. They show p53-driven nuclear exclusion of the forkhead box O1 (Foxo1) via posttranslational modifications by Sirt6 in the liver of mice, which led to decreased expression of phosphoenolpyruvate carboxykinase (*Pck1*) and glucose-6-phosphatase (*G6pc*) [100]. These contradictory results illustrate the complexity of studying p53’s context-dependent effects on metabolism. While there is strong evidence that p53 plays a major role in the adaption to food withdrawal in the liver, the effects of p53 on starvation in adipose tissue remain largely uncharacterized.

Studies performed in our laboratories showed that p53 is involved in the response to food withdrawal in epididymal WAT. By performing transcriptome analyses with concomitant functional annotation clustering of genes, we found 200 genes that are commonly upregulated under starvation conditions in three major metabolic tissues: epididymal WAT, liver, and skeletal muscle. Out of these genes, the p53 signaling pathway precipitated as central node in the regulation of starvation. We further investigated the role of the known p53-target [101] DNA damage inducible transcript 4 (*Ddit4*) that was induced under starvation in WAT, liver, and skeletal muscle. In differentiated C3H10T1/2 adipocytes, nutlin-3a activation of p53 increased *Ddit4* mRNA and protein levels and transient overexpression of *Ddit4* resulted in increased lipolysis [96].

A class of NAD^+^-dependent deacetylases, the sirtuins, are known as crucial energy sensors in many cells and tissues, with a special importance of Sirt1 [102]. Nemoto et al. reported that starvation-stimulated *Sirt1* expression by Foxo3a depends on a p53-Foxo3a interaction at the *Sirt1* gene promoter in rat adrenal gland cells [103]. In white adipocytes, Sirt1 has been shown to be crucial in the adaption to food withdrawal by activating fat mobilization. Additionally, Sirt1 binds to and represses target genes of Pparγ, thereby reducing adipogenesis [104], which is reminiscent of the inhibitory function of p53 in the adipogenic program. Therefore, it seems essential to investigate the p53-Foxo3a-Sirt1 axis directly in adipose tissue. Similar interactions between p53 and key nutrient sensing pathways, such as mTOR [105,106] and AMPK [107,108], reported mostly in cancer cells, could present worthwhile objects of experimental interrogation in adipose tissue.

Taken together, these studies propose an important role of the p53 signaling pathway upon hypercaloric stimuli as well as under starvation. Regarding nutrient deprivation as a profound stress stimulus, it seems feasible that p53, as a pivotal stress regulator, is activated and coordinates pathways essential to adapt to situations of acute nutrient scarcity. On the other hand, chronic nutrient abundance may result in chronic elevation of p53 signaling contributing to obesity and insulin resistance development [69,93]. Although the experimental settings for prolonged starvation both in cells and animals are difficult to establish, it would be intriguing to investigate p53’s levels and functions under short-term and long-term nutrient deprivation. Adding to the complex role of p53 in feeding behavior are results coming from two studies demonstrating an important role of p53 in the orexigenic action of ghrelin in the hypothalamus [109] and in the regulation of lipogenesis in WAT and liver in response to peripheral ghrelin treatment [110]. These findings put further emphasis on p53 as a central, if complex and tissue-specific, nutrient response factor.

## 5. p53 in Development, Maintenance, and Function of Brown Adipose Tissue and in Browning

Historically, BAT was believed to involute in postnatal humans, when the chronic demand for nonshivering thermogenesis decreases. More recently, however, ^18^FDG-PET-CT scans revealed that BAT exists in supraclavicular, neck, and paravertebral areas in adult humans (reviewed previously [111]). This was corroborated by several systematic studies in 2009 [112,113,114,115] that evoked widespread interest in the therapeutic potential of activating energy dissipation in BAT to curb diseases arising from energy surplus amounting to obesity and its associated metabolic pathologies. Together, this research has established a crucial role for BAT in whole body energy homeostasis [116] and several hallmarks of brown adipocyte development and maintenance, as well as for BAT function (reviewed previously [15,16]). Uncoupling protein 1 (Ucp1) is arguably the most prominent marker for BAT function as it is responsible for the energy dissipating aspect of the tissue via uncoupling cellular respiration from ATP synthesis in the inner mitochondrial membrane [117]. Other important findings in the field include the identification of myogenic factor 5 (Myf5)-positive BAT progenitor cells and of the transcriptional regulator PR domain containing 16 (Prdm16) as a marker of early brown adipocyte development [118,119]. The role of p53 in BAT function was interrogated by only few laboratories with partly overlapping and contradictory outcomes as discussed below.

In cultured cells, Molchadsky et al. showed that endogenous p53 levels are required for brown adipogenic differentiation of *Prdm16*-overexpressing myogenic C2 cells, as evidenced by a reduction of accumulated lipid droplets and lower expression of brown marker genes upon *p53* knockdown [45]. In contrast, brown differentiation efficiency of MEFs and primary adipocytes both derived from *p53* knockout mice was enhanced, evidenced by higher expression of *Ucp1* mRNA compared to their respective controls [54,120]. Accordingly, Hallenborg et al. reported that increased *Ucp1* expression correlated with higher isoproterenol-induced oxygen consumption rate in primary cells derived from inguinal white and interscapular brown depots of *p53* knockout mice [54]. The results of the latter two studies support a role of p53 as negative regulator of brown adipogenic differentiation from progenitor cells. This is consistent with p53 acting as a general suppressor of differentiation in embryonic stem cells and reprogramming in induced pluripotent stem cells [121], although they contrast the findings of Molchadsky et al. [45].

Data from different mouse models also paint a rather inconsistent picture that does not reflect the *in vitro* findings. First and foremost, although one study reported impaired BAT development in postnatal *p53* knockout embryos [45], data from mice with a p53 deletion specifically in *Myf5*-positive precursor cells demonstrated that *p53* expression in BAT progenitor cells is not necessary for normal development of BAT [122]. Furthermore, young, pre-leukemic whole-body *p53* knockout mice on a normal diet display no change in body weight, food intake, locomotor activity, or energy expenditure [45,54,122] when compared to wild-type controls. Only when fed a HFD, *p53* knockout mice showed decreased weight and fat mass gain and BAT *Ucp1* expression as reported in most studies [54,122,123] (a phenomenon apparently only existing in male, but not in female, *p53* knockout mice [123]). However, another report showed an increase in body fat and weight gain, accompanied by decreased *Ucp1* expression in BAT of *p53* knockout mice [45]. These discordant findings could be due to differences in housing conditions (e.g., thermoneutrality vs. hypothermia or group vs. single animal housing) and experimental timing, as discussed in Hallenborg et al. [54]. According to Al-Massadi et al. male *p53* knockout mice with partial resistance to HFD-induced weight gain showed increased energy expenditure despite equal food intake and locomotor activity. This was concomitant with increased BAT thermogenic activity and expression of BAT marker genes, such as *Prdm16* and adrenergic receptor 3 (*Adrb3*) [122]. Also, Ucp1 protein levels were elevated in BAT, altogether indicating BAT activation under global absence of p53 [54,122]. More tissue-specific *p53*-deficient models were employed by directly injecting adenoviruses expressing dominant negative *p53* into intrascapular BAT depots of chow-fed mice. In contrast to global *p53* knockout mice on HFD, body weight increased and BAT temperature and marker protein expression was reduced in this model, while all these parameters were reversed when injecting *p53*-overexpressing adenovirus into intrascapular BAT of HFD-fed mice [122]. In line with this, systemic activation of p53 with doxorubicin (also known as adriamycin) ameliorated weight gain under HFD possibly by increasing *Ucp1* expression in BAT, which was not apparent in *p53* knockout mice when treated with doxorubicin [122], therefore reflecting p53-specific actions of this drug. Thus, while whole body knockout of *p53* seems to activate BAT concomitant with reduced weight gain under HFD, direct manipulation of p53 levels specifically in interscapular BAT of mice indicate a positive correlation between p53 and weight gain, fat mass accretion, and BAT deactivation [122]. In the case of the whole-body knockout mouse, these differences could be explained by effects of p53 deficiency in other cells and tissues (e.g., in hypothalamic circuits that regulate feeding behavior and the sympathetic tone), that could cause a systemic shift in metabolic homeostasis towards energy dissipation in BAT.

Mechanistically, p53 seems to be involved in the regulation of the brown adipose phenotype by both protein–protein interaction and by transactivation of target genes. Protein–protein interaction between p53 and the transcriptional coactivator Pgc1α was suggested to mediate repression of Pgc1α-mediated regulation of *Ucp1* [54]. Furthermore, in a proteomics screen p53 was identified as interaction partner of Prdm16 in mouse brown adipocytes [124] and p53 was shown to bind to the *Prdm16* promoter in murine BAT [45]. Another intriguing mechanistic link is the above-mentioned nutrient-sensitive interaction of p53 and Foxo3a at the *Sirt1* promoter [103]. It will be important to determine whether the p53-Foxo3a-Sirt1 axis is also functionally relevant in brown and white adipocytes, because Sirt1 can be pharmaceutically targeted and is an essential determinant in the conversion of white to beige adipocytes [102]. p53’s role in this process of browning is less well-investigated, with the exception of two reports. In one, higher expression levels of genes involved in β-oxidation and of Ucp1 protein were observed in inguinal WAT of *p53* knockout mice [54]. The other report employed the WAT-specific and inducible Adipoq-creER/p53flox system to show that the age-dependent decline in cold-induced browning of inguinal WAT can be rescued by transient ablation of *p53*, which was concomitant with a reduction in mitophagy [125]. However, more work is needed to delineate how manipulation of p53 or its signaling could impact WAT browning. Moreover, global analyses to assess p53 binding to the brown adipocyte genome (i.e., with ChIP-seq) has not yet been performed and would aid to define p53’s transactivation role under different conditions, such as chronic or acute nutrient or temperature stresses.

Together, data from several mouse models indicate that p53 is not required for normal development of BAT and only becomes essential in chronic stress conditions such as the lipotoxic environment evoked by a hypercaloric diet. This is in agreement with p53 being activated by oleic acid [126] or palmitic acid [68,87] in cultured cells and suggests that p53 may be a central player in whole body energy homeostasis upon lipid overload, at least in part, through its action in BAT. However, it needs to be kept in mind that opposing data can be found in many cases, as described above. This calls for more research using defined experiments and read-outs in BAT-specific p53 models. For instance, inducible BAT-specific cre-expressing mice (Ucp1-CreER [127]) crossed with *p53*-floxed mice, would present a more precise platform to interrogate the role of p53 in BAT maintenance and function, as well as in whole body energy homeostasis.

## 6. Effects of p53-Interacting, p53-Inducible, and Transactivated Genes on Adipose Tissue Biology

**Figure 1:**
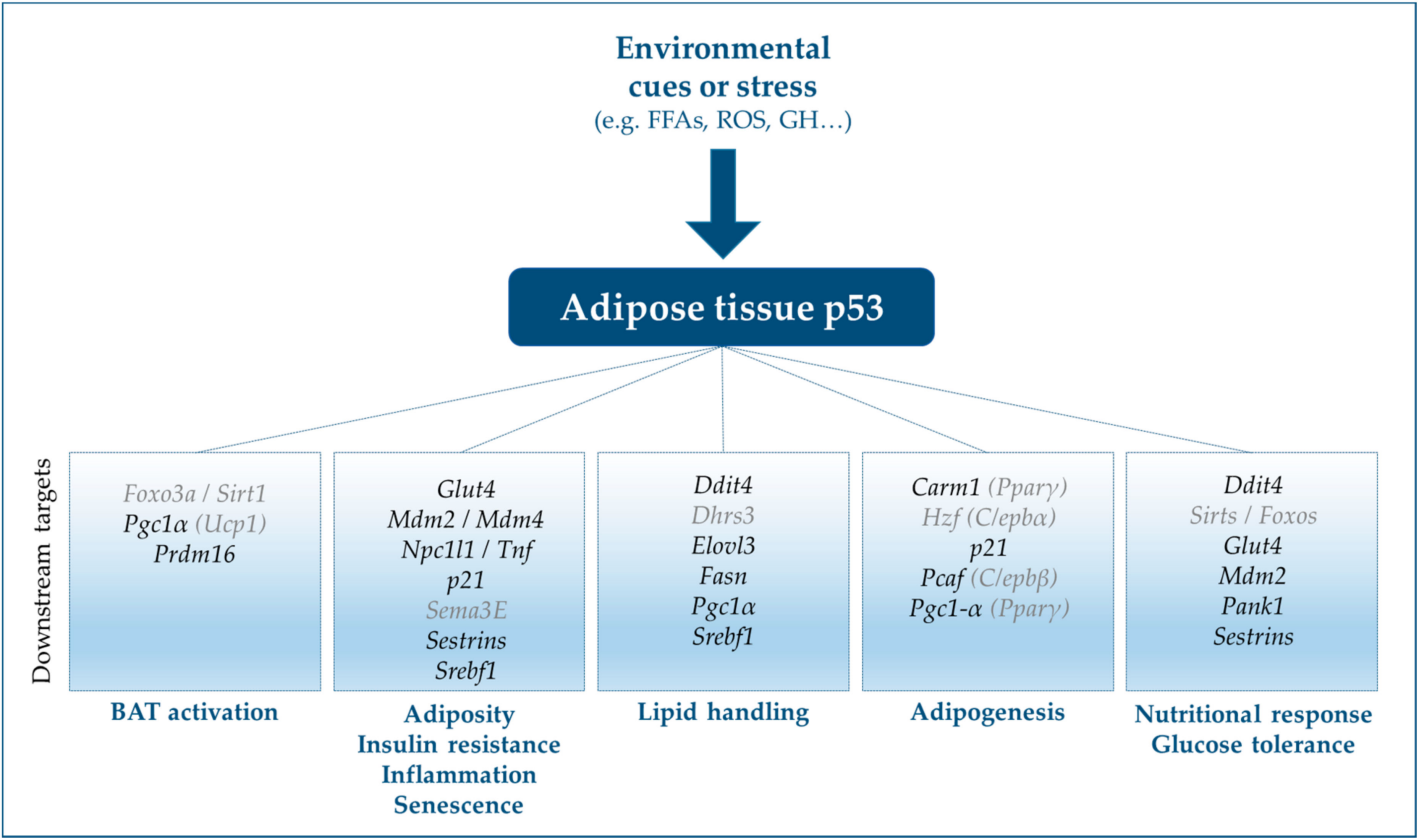
Actions of p53 through its target genes in adipose tissue. p53 levels in adipose tissue are determined by environmental cues and stress signals. By regulating expression of—or directly interacting with—its downstream targets, p53 coordinates several processes that are key in adipocyte development and maintenance and function of brown and white adipose tissue. Targets with a proven interaction between p53 and their promoters are in black letters, protein–protein interactions or p53-induced genes without evidence of direct regulation are in grey. In parentheses are key factors that are regulated by the corresponding p53 downstream targets.

Upon induction, the predominant modus operandi of the p53 protein is the reprogramming of the transcriptome by transactivation of a cell type-specific set of target genes [2,4,128]. Hence, it is likely that p53 exerts its effects in adipocytes and in adipose tissue via regulation of single or multiple target genes and their respective pathways (Figure 1). However, if p53 activity impacts on the expression of other transcription factors (e.g., zinc finger protein 385A (*Zfp385a* or *Hzf*)), coregulators, or chromatin modifiers, it can also exert indirect transcriptional effects via their respective target genes. Finally, it has also been shown that p53 can work through protein–protein interactions.

One example for direct target gene regulation with a relevance to metabolic homeostasis is the first discovered p53 target p21. *p21* knockout mice are phenotypically indistinguishable from littermate controls, but on a HFHS diet they gain less weight and show improved insulin sensitivity [58], thereby phenocopying these parameters in *p53* knockout mice (at least as reported previously [54,122]). Both WAT and BAT of *p21* knockout mice contain smaller adipocytes that are protected from high-caloric diet-induced hypertrophy. However, browning of WAT, energy expenditure, or thermogenic BAT activity were, to our knowledge, not reported for these models.

By acting as a macrophage-attractant, the p53 target Sema3e elicits aggravated adipose tissue inflammation and insulin resistance under HFHS diet, effects that were strongly reduced in Fabp4-Cre/p53-lox mice in which the lack of p53 in adipocytes led to reduced *Sema3e* expression and secretion [67].

Other well-established p53 target genes and at the same time (via a negative feedback loop) the most potent endogenous inhibitors of p53 [129], are *Mdm2* and *Mdm4* [4,130,131]. As mentioned in preceding chapters, mice with an *Mdm2* mutation that leads to reduced p53 activity showed reduced fat mass gain under a HFD (consistent with reports from *p21* [58] and *p53* [54,122] knockout mice) accompanied by increased energy expenditure [66], as also reported for *p53* knockout mice [122]. Interestingly, in cultured preadipocytes, Mdm2 seems to initiate adipocyte differentiation in a p53-independent manner by recruiting cAMP responsive element binding protein 1 (Creb1) to the *C/EBPδ* promoter [132]. Like with Mdm2, studying the physiological function of Mdm4 in whole body knockout mice has been impossible due to embryonic lethality. Crossed with *p53^3KR/3KR^* mice (an acetylation-defective *p53* mutant), however, *Mdm4* knockout mice are viable and exhibit resistance to HFD-induced fat accumulation. This was suggested to be mediated through mild p53 activation, followed by decreased WAT macrophage infiltration, increased lipid oxidation, and thermogenic activity of adipose tissues [133]. These results were largely consistent with data from adenoviral overexpression of *p53* in BAT or from pharmacological p53 activation [126]. In the *p53^3KR/3KR^/Mdm4*^−/−^ mouse model, elongation of very long chain fatty acids-like 3 (*Elovl3*) was identified as a direct p53 target gene that could mediate the observed effects on lipid handling [133].

As discussed above and reviewed previously [29], several p53 downstream factors impinge on other major hubs of adipocyte differentiation: *Pparγ* is regulated through interaction between its coactivator Pgc1α and p53 [46,134] and by the p53-target Carm1 [59]; *C/epbα* is regulated by the transcription factor *Hzf*, a p53-responsive gene [135]; C/EBPβ is acetylated by lysine acetyltransferase 2B (Kat2b or Pcaf), a known p53 target [130]. Together these data establish the p53 signaling network as important regulator for *in vitro* adipogenesis. Some evidence of a role of p53 in the maintenance and function of mature adipocytes is also available. For instance, our laboratories showed that the p53 target gene *Ddit4* [101] is increasing lipolysis if overexpressed in adipocytes differentiated from C3H10T1/2 cells [96]. Other examples are, *p21* knockdown in hypertrophied adipocytes (both *in vitro* and *in vivo*) interfering with adipocyte maintenance by inducing apoptosis [58] and the p53-inducible gene dehydrogenase/reductase member 3 (*Dhrs3*) being involved in lipid droplet budding at the endoplasmatic reticulum [136]. However, in cell types others than adipocytes, there is much more evidence of p53 as a central hub in lipid metabolism (as reviewed previously [28]).

In the light of the profound effects on adipose tissue and whole body energy metabolism exerted by p53 downstream factors, it seems imperative to continue to identify and investigate p53 targets in adipose tissue depots under metabolically challenging conditions. This is especially important in the search for pharmacological targets to treat metabolic diseases, since direct p53 manipulation could always lead to broad downstream effects and/or diminished tumor suppression, while therapeutically aiming at single downstream factors could evoke more focused treatment outcomes. 

## 7. Summary and Outlook

**Scheme 1:**
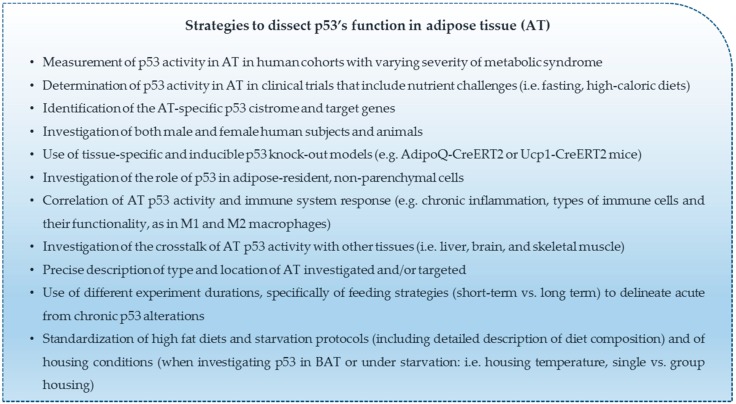
Strategies to dissect p53’s function in adipose tissue.

An effort to accurately recap the currently available literature about the role of p53 in adipose tissue biology is complicated by several factors (see Scheme 1).

First, there is a paucity of clinical data that evaluate p53 in adipose tissue in cohorts with varying degrees of metabolic syndrome or in clinical trials with acute and chronic nutrient challenges. Secondly, most data are available from whole body *p53* knockout mice. The observed metabolic changes cannot directly be attributed to adipose tissue and rather represent a cumulative effect of p53 deficiency in other cells and tissues such as hypothalamic centers that regulate feeding behavior [137]. Only a few studies manipulated p53 directly in adipose tissue [122] or employed adipose tissue specific knockout mice [67,68,71,125]. In the latter case, most studies used a system in which *p53* ablation was driven by the *Fabp4* promoter (Fabp4-Cre mice crossed with *p53* floxed mice). However, the *Fabp4* promoter was shown to be potentially active in tissues other than adipose tissues, such as macrophages and certain brain areas [138,139]. These inadvertent modifications of p53 levels in tissues other than adipose tissue may confound the data, especially when studying mechanisms of adipose tissue inflammation or systemic nutrient challenges. More specific mouse models are available by now, like systems based on the BAT-specific *Ucp1* promoter [127] or WAT-specific resistin (*Retn*) or *Adipoq* promoters [137,138]. Furthermore, tamoxifen-responsive inducible Cre systems have been developed that could help to investigate more acute and development-independent functions of p53 in adipose tissues [140,141], as shown in a recent study [125]. Thirdly, it would be essential to gain insight into the adipose tissue-specific p53 cistrome and the set of target genes regulated if certain stresses are prevalent (e.g., high levels of free FAs). Finally, it is important to note that some studies report contradictory results that we tried to reconcile throughout this review. However, evidence supported by the majority of published studies paint a picture of p53 as a crucial regulator of adipose tissue metabolism and homeostasis, especially when nutritionally challenged.
